# Effect of Different Lignocellulosic Diets on Bacterial Microbiota and Hydrolytic Enzyme Activities in the Gut of the Cotton Boll Weevil (*Anthonomus grandis*)

**DOI:** 10.3389/fmicb.2016.02093

**Published:** 2016-12-27

**Authors:** Emiliano Ben Guerrero, Marcelo Soria, Ricardo Salvador, Javier A. Ceja-Navarro, Eleonora Campos, Eoin L. Brodie, Paola Talia

**Affiliations:** ^1^Instituto de Biotecnología, Centro de Investigación en Ciencias Veterinarias y Agronómicas, Centro Nacional de Investigaciones Agropecuarias – Instituto Nacional de Tecnología Agropecuaria CastelarHurlingham, Argentina; ^2^Instituto de Investigaciones en Biociencias Agrícolas y Ambientales-Consejo Nacional de Investigaciones Científicas y Técnicas, Cátedra de Microbiología Agrícola, Facultad de Agronomía, Universidad de Buenos AiresBuenos Aires, Argentina; ^3^Instituto de Microbiología y Zoología Agrícola, Centro de Investigación en Ciencias Veterinarias y Agronómicas, Centro Nacional de Investigaciones Agropecuarias – Instituto Nacional de Tecnología Agropecuaria CastelarHurlingham, Argentina; ^4^Earth and Environmental Sciences, Lawrence Berkeley National LaboratoryBerkeley, CA, USA; ^5^Consejo Nacional de Investigaciones Científicas y Técnicas (CONICET)Buenos Aires, Argentina

**Keywords:** *Anthonomus grandis*, gut microbiota, 16S rRNA gene, illumina amplicon sequencing, hydrolytic activities, lignocellulosic feedstocks

## Abstract

Cotton boll weevils, *Anthonomus grandis*, are omnivorous coleopteran that can feed on diets with different compositions, including recalcitrant lignocellulosic materials. We characterized the changes in the prokaryotic community structure and the hydrolytic activities of *A. grandis* larvae fed on different lignocellulosic diets. *A. grandis* larvae were fed on three different artificial diets: cottonseed meal (CM), Napier grass (NG) and corn stover (CS). Total DNA was extracted from the gut samples for amplification and sequencing of the V3-V4 hypervariable region of the 16S rRNA gene. Proteobacteria and Firmicutes dominated the gut microbiota followed by Actinobacteria, Spirochaetes and a small number of unclassified phyla in CM and NG microbiomes. In the CS feeding group, members of Spirochaetes were the most prevalent, followed by Proteobacteria and Firmicutes. Bray–Curtis distances showed that the samples from the CS community were clearly separated from those samples of the CM and NG diets. Gut extracts from all three diets exhibited endoglucanase, xylanase, β-glucosidase and pectinase activities. These activities were significantly affected by pH and temperature across different diets. We observed that the larvae reared on a CM showed significantly higher activities than larvae reared on NG and CS. We demonstrated that the intestinal bacterial community structure varies depending on diet composition. Diets with more variable and complex compositions, such as CS, showed higher bacterial diversity and richness than the two other diets. In spite of the detected changes in composition and diversity, we identified a core microbiome shared between the three different lignocellulosic diets. These results suggest that feeding with diets of different lignocellulosic composition could be a viable strategy to discover variants of hemicellulose and cellulose breakdown systems.

## Introduction

Lignocellulosic ethanol has been proposed as a promising alternative to conventional fuel in the energy matrix. In fact, the lignocellulosic ethanol production is environmentally friendly and lignocellulosic feedstock is abundant. For these reasons, its production is considered to be sustainable. However, the production cost is high and requires a very efficient hydrolysis technology because of the recalcitrance of plant biomass.

Napier grass (NG; *Pennisetum purpureum* Schumach) is a potential biomass source for bioethanol production in Argentina and Brazil because it can be cultivated in marginal soils. NG is a perennial grass with a high growth rate (Lima et al., 2014; Ben Guerrero et al., 2015). Corn stover (CS), an abundant biomass residue in corn producing regions can also be used for the production of bioethanol (Kim et al., 2009; Whitman et al., 2011).

Lignocellulosic biomass and all plant cell walls are composed mainly of homo- and heteropolysaccharides, cellulose, hemicellulose and pectin, and a heterogeneous aromatic polymer, lignin. Cellulose degradation requires basically three types of synergistically acting enzymes: endoglucanases, exoglucanases and β-glucosidases. Likewise, xylanases are the main enzymes in the hydrolysis of hemicellulose. In addition, accessory enzymes (pectinases, arabinofuranosidases, mannanases, etc.) work in concert to break down the cell wall. Lignocellulose degradation in weevils depends on a dual system that includes activities of both the host and its intestinal symbionts (Calderón-Cortés et al., 2012).

*Anthonomus grandis* Boheman (Coleoptera: Curculionidae), which is also known as cotton boll weevil, is a serious cotton pest. Originally, this pest was found in Mesoamerica, but now extends from South Texas to Argentina. The population of boll weevils in subtropical and tropical regions is strongly related to the quality and distribution of different food sources throughout the year (Showler, 2007). Vegetative parts are the primary food during the cotton growing season, both for larvae and adults (Hunter and Pierce, 1912; Showler, 2002; Esquivel et al., 2004). However, during the off-season, around 5 months, *A. grandis* can fed on different nutritious food sources. For example, they can feed on flowering plants *Abutilon* sp., *Sphaeralcea* sp., *Sida* sp., and *Wissadula* sp. (Gaines, 1934; Stoner, 1968; Cross et al., 1975) and on the endocarps of citric fruits from prickly pear cactus, orange and grapefruit (Showler and Abrigo, 2007).

Many microorganisms participate in the degradation of lignocellulosic substrates and some of these microorganisms are present in the intestines and rumen of diverse animals. The knowledge of their enzymatic degradation system could contribute to broaden the sources of biocatalysts with biotechnological impact (Willis et al., 2010; Sun et al., 2013; Scharf, 2015; Batista-García et al., 2016).

There is extensive experimental evidence pointing out that the host diet shape the community structure and metabolic function of gut microbiota in different animals, including insects (Leser et al., 2000; Middelbos et al., 2010; Cardoso et al., 2012; Colman et al., 2012; Montagna et al., 2015). The existence of a core microbiota, which ensures the maintenance of the function, has been well documented in a variety of hosts.

Even though the role of the termite gut microbiota has been widely studied, little is known about the gut microbiota of weevils, their cellulolytic activities and adaptations to different diets (Bertino-Grimaldi et al., 2013; Schauer et al., 2014). In *A. grandis*, most studies have focused on bio-control strategies but their microbiota has been poorly assessed (Sikorowski, 1975; Hedin et al., 1978; Firmino et al., 2013; Salvador et al., 2014).

To investigate how the weevil microbiome responds to changes in diet and how the cellulosic activities were modified, we performed a screening by next-generation sequencing (NGS) of the bacterial communities present in the gut microbiome of *A. grandis* fed with three different lignocellulosic feedstocks. We sequenced amplicons covering the V3-V4 region of the 16S rRNA genes and complemented this data with a characterization of hydrolytic activities for each of the three diets. We hypothesize that the feeding habits determine microorganism abundance, which in turn affects the cellulolytic activities.

## Materials and Methods

### Chemical Composition of Lignocellulose Biomass

The contents of cellulose, hemicellulose and lignin in CS and NG were quantified gravimetrically according to the Neutral Detergent Fiber (NDF), Acid Detergent Fiber (ADF) and Acid Detergent Lignin (ADL) analyses (Goering and Van Soest, 1970). The proportions of cellulose, hemicellulose and lignin detected were 32, 2, and 5% respectively in CS and 46, 27, and 9% in NG.

Cottonseed meal (CM) is a commercial diet, rich in proteins (55%), 2% carbohidrates, 5% oil and 4% ashes (Archer Daniels Midland Company, cat # 069059). This diet, called Pharmamedia, and contains 9.6% cellulose, 8.7% hemicellulose and 5.4% lignin.

Pectin content was determined according to Fry (1988). Dry samples were extracted with ethanol (66 mg/mL) at 80°C for 2 h. The alcohol insoluble residue was extracted with hot water (15 mg/mL, 90°C, for 3 h) to get a starch free residue (CW). CW was extracted twice at room-temperature with 0.05 M Na_2_CO_3_, containing 20 mM NaBH_4_. Both extracts were combined, dialyzed against distilled water twice and finally lyophilized. This procedure was carried out by triplicate and pectin content was expressed as percentage of material extractable with dilute sodium carbonate solution. These values were 10% in CM, 4% in CS and 2% in NG.

### *Anthonomus grandis* Boheman Rearing on Artificial Diets

*Anthonomus grandis* larvae were reared at the Institute of Microbiology and Agricultural Zoology (IMYZA), INTA. The larvae were raised on three different autoclaved lignocellulosic artificial diets: CM, CS and mature NG whole plants at 28°C, 70% relative humidity, with a 12-h light/darkness photoperiod. The larvae were maintained on each diet for 10 days, a period sufficient for *A. grandis* to grow throughout three developmental stages. After 10 days, the larvae were surface disinfected with 70% ethanol and dissected under a binocular microscope. Ten dissected guts were taken from a single plate, grounded in bidistilled water, homogenized by vortexing and centrifuged at 12,000 *g* for 10 min at 4°C. Three independent plates were used per treatment. A Protease Inhibitor Cocktail Kit (Thermo Scientific, USA) (1 μl/mL) was added to the supernatant and this supernatant was stored at -20°C for enzymatic assays.

### DNA Extraction and Bacterial 16S rRNA Gene Library Construction

Microbial genomic DNA was extracted from three gut samples using the DNeasy Blood and Tissue kit (Qiagen, USA) according to the manufacter’s instructions. The V3-V4 hypervariable regions of 16S rRNA gene were amplified in triplicate from microbial genomic DNA using the following universal primers: 515F (5′-GTGCCAGCMGCCGCGGTAA-3′), (Turner et al., 1999) and 806R (5′-GGACTACHVGGGTWTCTAAT-3′), (Caporaso et al., 2012) with sequencer adapters and sample-specific Golay barcodes on the reverse primer. Polymerase chain reactions (PCR) were performed in triplicate per sample by using 1 × Takara ExTaq PCR buffer with MgCl_2_, 10 μM of primers, 0.56 μg/μL bovine serum albumin, 200 μM dNTPs, 0.025 U ExTaq DNA polymerase (Takara Mirus Bio Inc., Madison, WI, USA), 10 ng template and milliQ H_2_O to complete 25 μL volume. PCR cycling was performed with an initial denaturation at 95°C for 3 min, followed by 30 cycles at 95°C for 30 s, annealing at 55°C, for 45 s, extension at 72°C for 90 s and a final extension of 72°C for 12 min. The triplicate reactions were pooled and purified using SPRI magnetic beads (Agencort AMPure XP, Beckam Coulter Inc. Brea, CA, USA) (1.2 × μL of sample volume) to select for 400 bp amplicons according to the manufacturer’s protocol. The samples were then quantified using a Qubit^®^ fluorometer (Qiagen). Concentrations of each sample were calculated and then diluted to 10 nM. All samples were pooled in equimolar amounts for sequencing.

The amplicons were sequenced using the Illumina MiSeq kit v3 and 300 PE sequencing cycles.

### Sequence Analyses and Taxa Identification

The initial processing and quality control of the paired-end reads was performed using Mothur (Schloss et al., 2009) following the SOP developed by Kozich et al. (2013). The main steps were merging the 250-bp ends of each paired-end read into a single sequence with a quality-aware algorithm. Sequences with any number of ambiguities as well as those longer than 265-bp were removed. The remaining sequences were aligned against the Mothur-adapted SILVA reference alignment (Quast et al., 2013)^1^^,^^2^. Those sequences that started after alignment position 13,862 or ended before position 23,444 and those with homopolymers longer than eight bases were also removed. This procedure was followed by a noise reduction step using the precluster algorithm and the removal of chimeras with the UCHIME algorithm, both as implemented in Mothur. Finally, the sequences were given a taxonomic classification with Mothur’s Bayesian classifier and the SILVA reference database release 123 (Wang et al., 2007). OTUs were clustered for each sample before and after removing of singletons and doubletons. Less than 0.2% of the sequences were classified as Archaea and they were therefore removed.

The experiment consisted of three treatments, each with three biological replicates. From the nine samples we obtained 72,028 high-quality sequences reads. However, there were large differences in reads per sample, ranging from 4,400 to 26,633. In consideration to these variations, all subsequent analyses were performed on normalized subsamples of 4,400 reads (39,600 reads, ∼55% of the initial reads) and 3,747 for analyses excluding rare sequences (singletons and doubletons).

The cleaned and normalized dataset was used to cluster the sequences into taxonomic operational taxonomic (OTUs) units with a similarity cutoff of 0.97.

The observed species richness was defined as the number of OTUs present in each sample. Also the Chao’s richness estimator and the Simpson’s inverse index of diversity were calculated using Mothur (Schloss et al., 2009). The patterns of OTUs diversity were examined using rarefaction curves.

Weighted Unifrac and Bray–Curtis dissimilarity matrices were built for β-diversity (inter community) analyses. To represent the data graphically, we performed a NMDS analysis. The UniFrac distances among microbiomes were tested with a permutation test. The differences among diets in the Bray–Curtis matrix distance were tested with the Analysis of Molecular Variance (AMOVA) test (Anderson, 2001). Heat maps were constructed using the program MeV v.4.8.1 for Windows, TM4 Software (Saeed et al., 2003).

All sequence data was deposited in the NCBI Sequence Read Archive under the BioProject accession number PRJNA327396.

### Enzymatic Activity Assay in *A. grandis* Fluids

*Anthonomus grandis* gut extract (GE) samples (20–30 guts each) from third instars were analyzed using dinitrosalicylic acid (DNSA) assay for determination of reducing sugar. Three biological replicates (gut extracts) were done per treatment and for each biological replicate the determinations were repeated three times. Under these conditions both endogenous and exogenous glycosyl hydrolases are detected. The reaction was adapted to small volumes (King et al., 2009) and for the hydrolysis of carboxymethyl cellulose (CMC), xylan and pectin. The assays were performed using 100 μg of GE and 100 μl of either 1% CMC, 1% xylan or 1% pectin, in 0.1 M phosphate citrate buffer (pH 4 and 5) or 1 M sodium phosphate buffer (pH 6–8) and incubated at 50°C for 60 min (CMC) or 30 min (xylan and pectin). Gut extracts without substrate and with the substrate in buffer without enzymes served as negative controls. A commercial cellulase from *Aspergillus niger* (Sigma, USA) was used as a positive control. Absorbance readings at 540 nm were compared to standard curves prepared with glucose, xylose or D-galacturonic acid ranging from 0.05 to 2.5 mg/mL. The enzyme activity (U/mL) was determined considering 1 IU equivalent to 1 μmol of glucose, xylose or D-galacturonic acid released per min under the assayed conditions.

The β-glucosidase activity was measured with 4-Nitrophenyl β-D-glucopyranoside (*p*NPG, Sigma, USA) as a cellobiose analog. The reaction assay was performed as follows: 100 μg of GE were incubated with 100 μL of 5 mM *p*NPG in 0.1 M phosphate citrate buffer (pH 4 and 5) or 1 M sodium phosphate buffer (pH 6–8). After 30 min, the reaction was stopped by adding 500 μL of 0.2% sodium carbonate and the absorbance was measured at 400 nm. A standard curve was prepared with *p*-nitrophenol (*p*NP). One unit (U/mL) of β-glucosidase activity was defined as the amount of enzyme that released 1 μmol of *p*NP per min under the assayed conditions.

The protein concentration was measured by the bicinchoninic acid assay (BCA, Thermo Scientific, USA), with bovine serum albumin (BSA) as a standard.

Data were expressed as the mean ± one standard deviation of the biological triplicates measurement. Enzymatic activity data were analyzed for statistical significance by a two-way analysis of variance (ANOVA) and post-test Tukey’s multiple comparison using GraphPad Prism 6 for Windows, GraphPad Software (San Diego, CA, USA).

### Effect of pH and Temperature on Hydrolytic Activity

The effects of pH and temperature on the enzyme activity of gut extracts from each diet were estimated at five pH values (4.0, 5.0, 6.0, 7.0, and 8.0) and three temperatures (30°C, 50°C, and 80°C) by triplicate. The buffers used were 0.1 M phosphate citrate (pH 4 and 5) and 1 M sodium phosphate (pH 6–8). The assays were performed as described above.

## Results

### Bacterial Diversity

**FIGURE 1:**
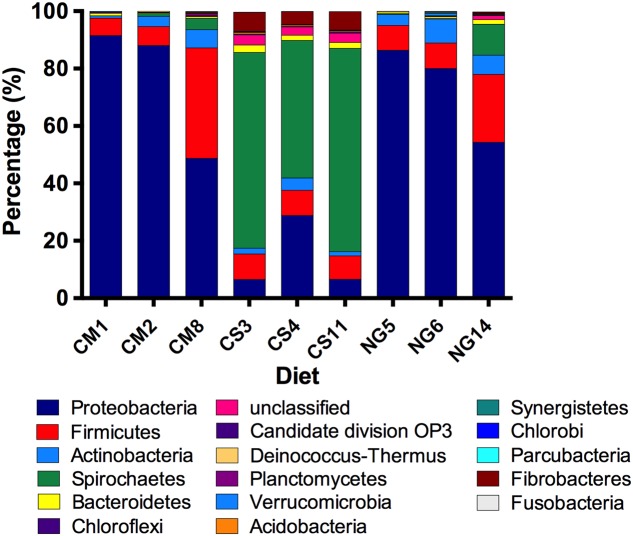
**Relative abundance of bacterial phyla in the gut of *A. grandis* reared in cottonseed meal (CM), CS and Napier grass (NG) artificial diets**. The V3-V4 hipervariable regions of the 16S rRNA gene were sequenced using the Illumina Miseq platform in independent triplicate samples for each diet. Amplicons were assigned a taxonomic identification and quantified. With varying proportions, Protebacteria, Firmicutes and Spirochaetes were the dominant phyla in all samples. Bacterial phyla at a sequence abundance of 0.1% or higher are shown.

We detected 15 bacterial phyla and a low number of unclassified bacteria across diets (**Figure 1**). Within the CM group, the two dominant phyla were Proteobacteria (76% of reads) and Firmicutes (17%), followed by Actinobacteria (4%) and Spirochaetes (2%). We also detected Bacteroidetes, Deinococcus-Thermus, Chloroflexi and Candidate_division_OP3 with abundances between 0.1 and 1%. Around 0.2% of the reads corresponded to unclassified bacteria. The NG group was also dominated by Proteobacteria and Firmicutes followed by Actinobacteria, Spirochaetes, Bacteroidetes, Verrucomicrobia Deinococcus-Thermus, Acidobacteria, Fibrobacteres, Fusobacteria, Parcubacteria and Planctomycetes (74, 14, 6, 4, 1, 0.3, 0.2, 0.1, 0.1, 0.1,0.1, 0.1% of the reads, respectively). One percent of the reads remained unassigned to any phylum with this analysis. Noteworthy, in the CS feeding group, the most predominant group was Spirochaetes (62%), followed by Proteobacteria (14%), Firmicutes (9%), Fibrobacteres (6%), Actinobacteria (3%), Bacteroidetes (2%) and Acidobacteria (0.5%), Planctomycetes (0.3%), Chlorobi (0.2%) and Synergistetes (0.1%). Three percent of the reads belonged to OTUs that remained outside any phylum classification.

### Biodiversity and Richness Estimates

**FIGURE 2:**
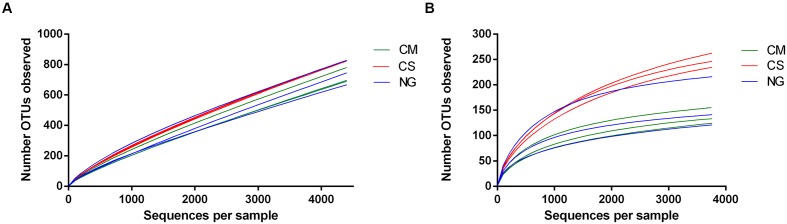
**Rarefaction curves of OTUs (clustered at 97% sequence identity) of the bacterial communities in the gut of *A. grandis* larvae fed with three artificial diets: cottonseed meal (CM), CS and NG. (A)** Number of distinct OTUs counted at each rarefaction step **(B)** Rare OTUs were removed (singletons and doubletons) and the rarefaction curves were built as in **(A)**.

We then calculated the rarefaction curves of OTUs defined at 97% similarity for all OTUs and OTUs that had at least three reads (that is, excluding singletons and doubletons, or very rare OTUs). After removing rare OTUs (**Table 1**), we obtained high coverages (>0.98) for all samples. This finding suggests that the sample size was large enough to represent the bacterial diversity of non-rare OTUs present in the communities from weevils fed with CM, CS and NG diets (**Figure 2**).

**Table 1:** Richness estimate and diversity index for gut samples under different diets^a^.

Group	With rare sequences				Without rare sequences			
		Richness	Diversity			Richness	Diversity	
		
	Obserted	Chao	InvSimpson	Coverage	Observed	Chao	InvSimpson	Coverage
CM1	696	6913.2 (4911–9867.1)	7.7 (7.3–8.1)	0.86	125	181.4 (151–248.1)	5.8 (5.5–6)	0.99
CM2	690	5849 (4250.4–8164.4)	9 (8.5–9.5)	0.86	134	156.9 (144–186.6)	6.8 (6.5–7.2)	0.99
CM8	780	5860.1 (4390–7929)	14 (13.2–15)	0.86	155	223.3 (185.1–310)	10.2 (9.6–10.8)	0.99
CS11	823	3427.6 (2812–4234)	13.7 (12.8–14.7)	0.85	235	273.3 (256–315.3)	10.2 (9.6–10.7)	0.98
CS3	826	3271.3 (2692.8–4029.2)	14.7 (13.8–15.8)	0.86	147	283.9 (267–315.3)	11 (10.3–11.7)	0.98
CS4	828	3892 (3137.4–4892.6)	22.9 (23.4–26.6)	0.85	264	336.2 (307–386.2)	18.8 (17.7–20)	0.98
NG14	828	5094.3 (3910.3–6733.2)	22.2 (20.8–23.8)	0.86	216	261.3 (237–314.4)	16.2 (15.3–17.3)	0.99
NG5	745	6853.5 (4993.4–9528)	11.3 (10.7–12)	0.85	121	158 (137.3–205)	8.3 (7.8–8.7)	0.99
NG6	667	5361.5 (3869–7550)	13 (12.3–13.8)	0.88	142	173 (155–217.4)	10 (9.5–10.6)	0.99

*^a^Diversity and richness indices were estimated based on 3% differences in nucleic acid alignment before and after removing rare sequences. Values in parenthesis are 97% of confidence intervals and were calculated by Mothur (Schloss et al., 2009) program*.

The bacterial communities obtained from CS showed the higher richness. The diversity, as measured by the Simpson’s inverse index, was also higher in the CS community; however, the difference with the NG diet was not so marked, in contrast to what was observed for richness (**Table 1**). On the other hand, the CM community was the less rich. The community obtained with this diet also showed a lower Simpon’s diversity index, thus indicating that it was the most uniform community.

### Diversity between Communities

**FIGURE 3:**
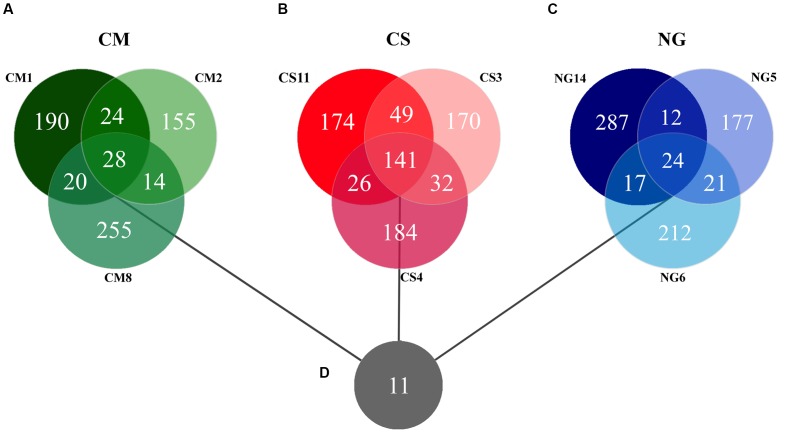
**Venn diagrams of the triplicates for each artificial diet and overall core**. The Venn diagrams for each diet show the unique OTUs and those shared by two or three replicates of each artificial diet, **(A)** Cottonseed meal, **(B)** Corn stover, **(C)** NG. **(D)** Depicts the overall core of 11 OTUs. The genetic distance cutoff for OTU definition was set at 0.03.

**FIGURE 4:**
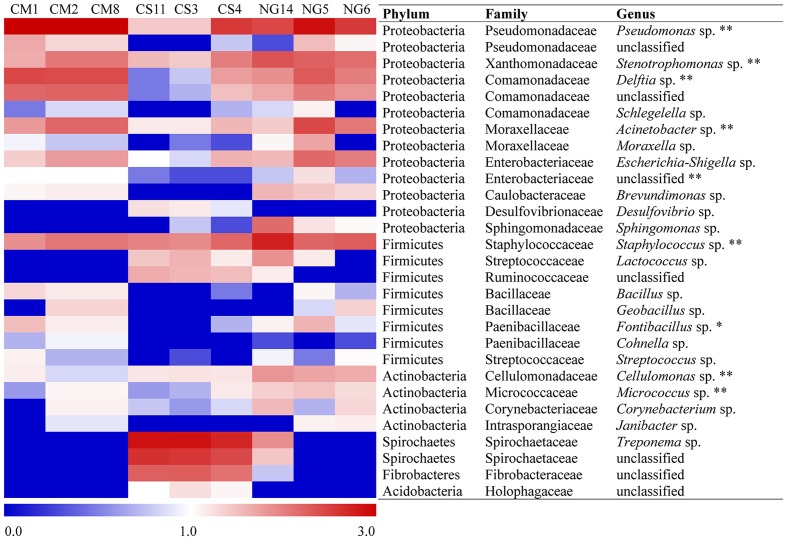
**Relative abundance of the most-abundant bacteria taxa in the gut of *A. grandis* across cottonseed meal (CM), corn stover (CS) and Napier grass (NG) artificial diets**. Classification is shown down to the genus level. The heat map was constructed with logarithmic counts to facilitate the visualization of low abundant groups. Redder and bluer colors indicate higher and lower abundances, respectively. A single asterisk indicates bacteria present in the core, and double asterisks, cellulolytic bacteria present in the core.

The patterns of co-occurrence of OTUs in the microbiomes observed with the different diets were depicted using Venn diagrams. We found that only 28 out of 686 OTUs were shared among the three replicates in gut extract of *A. grandis* fed in CM (**Figure 3A**). For the CS communities, the numbers of shared OTUs was higher, 141 out of 776 total OTUs; however, a significant number of OTUs seemed to be unique to each replicate (**Figure 3B**). Finally, we obtained similar results for the NG communities; only 24 out of 750 OTUs were shared among the triplicate (**Figure 3C**, Supplementary Table S1). When we repeated the analysis omitting the rare OTUs, we obtained similar results in the shared OTUs for each diet. However, the number of unique OTUs per diet decreased (data not shown). Eleven OTUs were shared between the three different diets, which were assigned to three phyla, ten families and eight genera (**Figure 3D**, **Table 2**). These shared OTUs belong to the most abundant genera and showed strong variations in abundance among diets and replicates of the same diet. Seven of these shared OTUs are members of the group of the eight most abundant cellulolytic genera detected in this survey: *Delftia* sp., *Acinetobacter* sp., *Stenotrophomonas* sp., *Staphylococcus* sp., *Cellulomonas* sp., *Pseudomonas* sp. and *Micrococcus* sp. (**Figure 4**).

**Table 2:** Shared OTUs of cotton boll weevils fed with CM, CS and NG artificial diets.

OTUs	Phylum	Family	Genus
0001	Proteobacteria	Enterobacteriaceae	Unclassified
0002	Proteobacteria	Pseudomonadaceae	*Pseudomonas* sp.
0004	Proteobacteria	Comamonadaceae	*Delftia* sp.
0008	Proteobacteria	Xanthomonadaceae	*Stenotrophomonas* sp.
0014	Proteobacteria	Moraxellaceae	*Acinetobacter* sp.
0015	Proteobacteria	Moraxellaceae	*Acinetobacter* sp.
0144	Proteobacteria	Enterobacteriaceae	Unclassified
0006	Firmicutes	Staphylococcaceae	*Staphylococcus* sp.
0007	Firmicutes	Panibacillaceae	*Fontibacillus* sp.
0011	Actinobacteria	Cellulomonadaceae	*Cellulomonas* sp.
0022	Actinobacteria	Micrococcaceae	*Micrococcus* sp.

### Analysis of Community Structure

**FIGURE 5:**
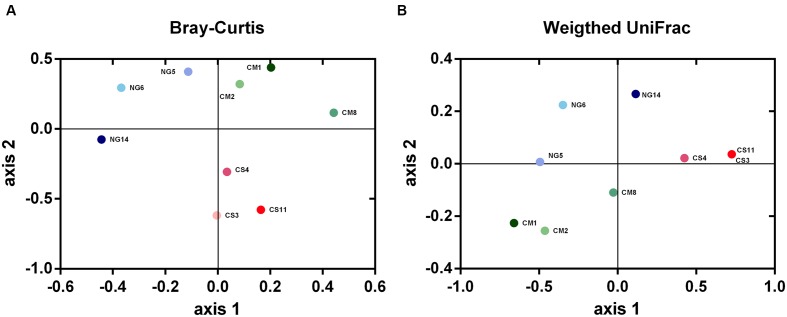
**Nonmetric multidimensional scaling (NMDS) plots derived from pairwise Bray–Curtis (A)** and weighted UniFrac **(B)** distances between bacterial communities from *A. grandis* gut fed with different artificial diets. Both distances showed significant differences across diets when analyzed with permutation-based tests (*P* = 0.011 and *P* < 0.005 for the Bray–Curtis and Unifrac distances, respectively). The triplicate gut communities from the corn stover diet (CS, pink) were clearly separated from those of cottonsead meal (CM, green) and Napier (NG, blue) diets.

The distances among bacterial communities based on OTUs present and their abundances were represented using a Bray–Curtis dissimilarity matrix and visualized with NMDS plots. These analyses revealed that the bacterial community in guts of CS-fed *A. grandis* was clearly separated from those of CM and NG diets on the second ordination axis. Besides, The NG and CM bacterial communities were separated on the second axis (**Figure 5A**). A similar pattern was observed with a weighted UniFrac dissimilarity matrix but the separation between CM and NG communities was less clear on the first axis (**Figure 5B**). A permutation-based test on the weighted UniFrac distances showed significant differences between artificial diets (*P* < 0.005), although no differences were found with unweighted Unifrac distances. This result suggests that the differences in communities were due to differences in relative abundances, and not so much to differences in OTUs identity. In addition, we carried out an AMOVA test on the Bray-Curtis matrix distance of all replicates and treatments. This analysis, also showed significant differences among diets (*P* = 0.011).

### Characterization of Hydrolytic Activities across Different Diets

**FIGURE 6:**
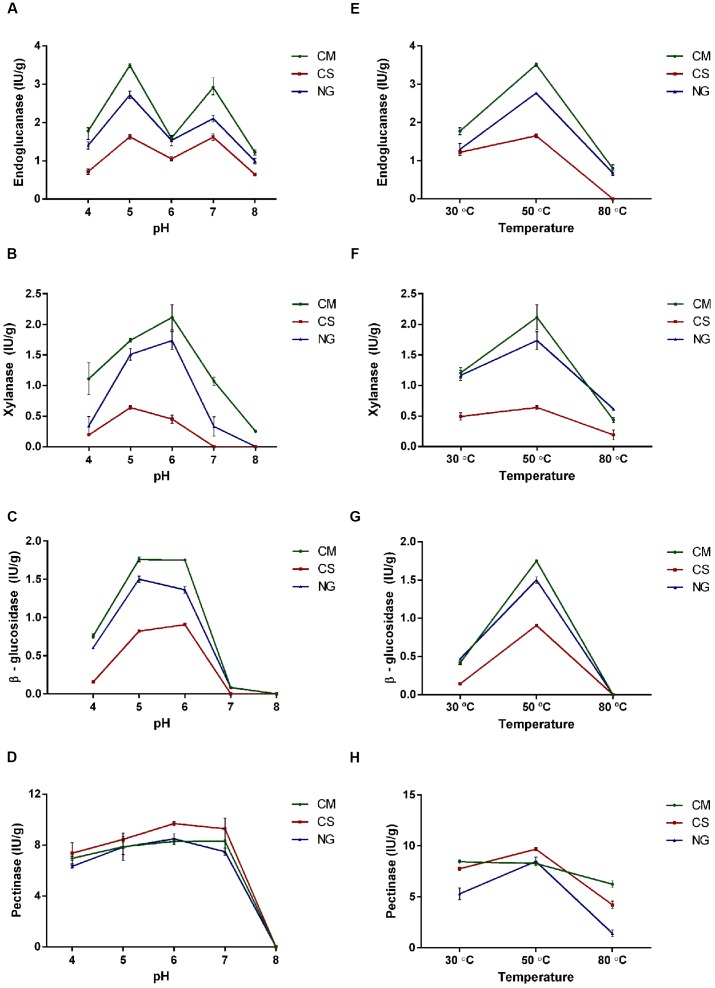
**Characterization of endoglucanase, xylanase, β-glucosidase and pectinase activities under different pH and temperatures in gut extract of *A. grandis* larvae fed with three artificial diets [cottonseed meal (CM), corn stover (CS) and Napier grass (NG)]. (A,E)** Endoglucanase, **(B,F)** Xylanase, **(C,G)** β-glucosidase, **(D,H)** Pectinase. Data were calculated as the mean ± one SD of biological triplicate measurements. Enzymatic activity data were analyzed for statistical significance with a two-way ANOVA and post-test Tukey’s multiple comparison.

We compared endoglucanase, xylanase, β-glucosidase and pectinase activities in entire guts of third instar larvae fed with three artificial diets: CM, CS and NG. Larvae reared on the CM diet showed significantly higher activities of endoglucanase, xylanase and β-glucosidase compared with the two others (*P* = 0.0001, **Figure 6**).

We then characterized the enzymatic activities under five levels of pH in the range 4.0–8.0, and three temperatures (30, 50, and 80°C). We found that the endoglucanase activity on the three diets exhibited two optimal pH peaks at 5 and 7 (**Figure 6A**).

The xylanase activity showed a pH dependence that was in turn influenced by diet (**Figure 6B**). Under the CM diet, this activity was significantly higher (*P* < 0.0001) and peaked at pH 6. With the NG diet, the profile was flatter. On the other hand, with the CS diet the activity was distinctly lower and peaked at pH 5 with a slight reduction at pH 6.

The optimal pH for the β-glucosidase activity was between pH 5–6 for CM, pH 6 for CS and pH 5 for the NG. Above pH 6, the β-glucosidase activity was completely lost (**Figure 6C**).

The pH characterization of the pectinase activity showed the highest activity in the range of 5–7 for the three diets, with no detectable activity at pH 8. This enzyme was the less affected by the type of diet (**Figure 6D**).

The optimal temperature was 50°C for endoglucanase with the three diets. This temperature was also the optimal for xylanase and β-glucosidase activities under CS and at pH 5; and for pectinase activity under CM and NG diets and at pH 6 (**Figures 6E–H**).

## Discussion

*Anthonomus grandis*, as other insects, harbors endogenous and symbiotic microbiota enzymes capable of breaking down the plant cell wall. Today, *A. grandis* gut transcriptome has been completely sequenced (Firmino et al., 2013; Salvador et al., 2014). In addition, several endogenous glycosyl hydrolases were identified (Salvador et al., personal communication) but the relationship between the host and its gut microbiome regarding the degradation of complex cell wall components remains unclear. In particular, the response of the gut microbiota to changes in diets has not yet been studied.

Host diet shapes the community structure and metabolic function of gut microbiota in different animals, including insects (Leser et al., 2000; Middelbos et al., 2010; Cardoso et al., 2012; Colman et al., 2012; Montagna et al., 2015). In termites, several studies have focused on how the dietary specialization determines the composition of the gut microbiota, and proposed that host diets shape termite gut microbiomes (Colman et al., 2012; Mikaelyan et al., 2015; Rahman et al., 2015). Indeed, Montagna et al. (2015) found that food sources significantly influenced the bacterial community in *Rhynchophorus ferrugineus* (red palm weevil). Furthermore, Colman et al. (2012) studied the influence of diets on gut bacterial communities of 58 insect species. Their work revealed that dietary effects are more evident in insects fed with lignocellulose material.

The changes in rarefaction curves and diversity indices herein described suggest that diets induce large changes in the structure of the microbial communities, regarding richness, diversity and taxonomical composition. *A. grandis* fed with CS showed higher bacterial diversity and richness than with the other two diets, with an important decrease in Proteobacteria and an increase in Spirochaetes phyla. Interestingly, when we removed rare sequences from the analysis, the diets effects were even more evident.

About 90% of the phyla found in whole larvae gut extracts fed with CM and NG artificial diets were Proteobacteria and Firmicutes. On the other hand, an important decrease in Proteobacteria occurred with CS diets; in this case, we could assign a high number of Spirochaetes OTUs. The same predominant groups have been previously reported in other weevils (*Rhynchophorus ferrugines* and *Rhynchophorus Vulneratus*), beetles (*Pachisoma endroedyi*, *Pachisoma striatum*, *Megetra cancellata*, *Epicauta longicollis*, *Gonasida inferna* and *Calosoma peregrinator*), cotton leafworm (*Spodoptera littoralis*), higher termites (*Nasutitermes aquilinus and Cortaritermes fulviceps*), honey bee (*Apis mellifera*), planthopper (*Lycorma delicatula*) and yellow ladybird (*Illeis koebelei*) (Colman et al., 2012; Shao et al., 2014; Yun et al., 2014; Ben Guerrero et al., 2015; Montagna et al., 2015; Franzini et al., 2016). Furthermore, Spirochaetes have been reported previously as a dominant component of the termite gut microbiota (Warnecke et al., 2007; Husseneder et al., 2010; Boucias et al., 2013; Benjamino and Graf, 2016). These changes could be due to differences in diet heterogeneity; more diverse diets with greater complexity of nutrients, such as CS, may require a more diverse bacterial group (Bertino-Grimaldi et al., 2013). In addition, the gut microbiota of non-social insects, such as *A. grandis*, is acquired from the environment. In this context, a significant variation between insects may occur. Similar variations were also observed in other gut communities (Thompson et al., 2008; Husseneder et al., 2010; Boucias et al., 2013; Schauer et al., 2014). Additionally, 16S rRNA gene surveys revealed that lignocellulosic diet shifts have no short-term impacts on microbiota composition in social insects such as termites and cockroaches (Sanyika et al., 2012; Boucias et al., 2013; Schauer et al., 2014).

In addition, the ordination of the communities on a NMDS plot based on Bray–Curtis and Unifrac distances showed the CS community was clearly separated from those of CM and NG diets. Our study revealed significant variation in the community structure of *A. grandis*, both between samples and between replicates of the same diet groups. These variations between biological replicates have been observed previously. Indeed, Curtis and Sloan (2004) postulated that microbial communities of physically identical environments will differ in composition when they are formed from a large and diverse group of microorganisms. Other insects (termites and cockroach), land snails, goats, pigs and humans showed similar variations in their gut communities (Ley et al., 2006; Thompson et al., 2008; Cunha et al., 2011; Cardoso et al., 2012; Bertino-Grimaldi et al., 2013; Schauer et al., 2014; Mikaelyan et al., 2015).

A small number of OTUs were present in all replicates of the three diets. However, these OTUs represent about 27% of all the obtained sequences. In general, they ranked among the OTUs that tended to show a high variability in their read counts across samples. Some authors proposed that some host species, especially those consuming highly variable diets, have a core microbiota that provides functional stability and sustain gut homeostasis (Turnbaugh et al., 2009; Qin et al., 2010; Otani et al., 2014; Schauer et al., 2014). These functional services can be satisfied by different taxonomic entities. In line with this notion, some authors proposed the existence of functional and taxonomic cores (Turnbaugh et al., 2009; Qin et al., 2010; Schauer et al., 2014). Of all the distinct OTUs detected in the replicates per diets, only small fractions were part of their respective cores. At this point we are not certain about the functional importance of these cores (**Figure 3**). In some insects, the core microbiota is small; only two, nine, ten and fifteen OTUs taxa have been identified in *Anopheles gambiae*, *Apis mellifera* and *Pachysoma* sp., *Cimex lectularius* and *Rhynchophorus* sp., respectively (Wang et al., 2011; Moran et al., 2012; Sabree et al., 2012; Meriweather et al., 2013; Montagna et al., 2015; Franzini et al., 2016).

The 11 OTUs of the overall core belong to some of the most abundant genera (**Table 1**; **Figure 4**). *Lactococcus* sp., *Bacillus* sp., *Brevundimonas* sp. and *Corinebacterium* sp. were also reported as the most abundant genera in the weevil *Rhynchoporus ferrugines* olivers (Tagliavia et al., 2014; Montagna et al., 2015). The eleven OTUs mentioned above are members of families already reported in insect microbiomes (Hedin et al., 1978; Campbell et al., 1992; Priya et al., 2012; Prabhakar et al., 2013; Montagna et al., 2015; Xu et al., 2015; Franzini et al., 2016).

We postulate that the bacterial gut communities of *A. grandis* respond to diet changes by maintaining a stable core with simultaneous variations in the presence of other OTUs. Whether these additional OTUs are opportunistic commensalists, functionally equivalent symbiotic mutualist or a mixture of both, remains to be established. It is noteworthy that in insects fed with the CS diet, the gut microbiome is very different to that observed with other diets and mainly comprises Spirochaetes bacteria.

We are especially interested in identifying genera with potential lignocellulose activity. These genera could be part of the core or abundant non-core genera that are functionally redundant, so that they can be replaced by alternative taxa. In this way, the microbiome carries all the required degrading capabilities contributed from core or non-core functionally substitutes OTUs. In the overall core, we found seven genera for which lignocellulose degrading capabilities were already reported (*Acinetobacter* sp., *Delftia* sp., *Stenotrophomonas* sp., *Micrococcus* sp., *Staphylococcus* sp., *Cellulomonas* sp. and *Pseudomonas* sp.). The genus *Acinetobacter* was present in the gut of the termite *Cortaritermes fulviceps* (Ben Guerrero et al., 2015), in the midgut and haemolymph of *Leptinotarsa decemlineata*, *Microcerotermes diversus*, and a in the gut of the giant African snail (*Archachatina marginata*). Their cellulase, xylanase and β-glucosidase activities were quantitatively evaluated (Ekperigin, 2007; Pourramezan et al., 2012; Vilanova et al., 2012). *Delftia* sp. was described as forming part of cellulolytic communities in several ecological niches (Juárez-Jiménez et al., 2010), including soils (Mehnaz et al., 2010). This genus was also isolated from a filter paper culture of native soil (Talia et al., 2012). Several researchers reported *Stenotrophomona* sp. as cellulolytic (Huang et al., 2012; Talia et al., 2012; Pinheiro et al., 2015). In addition, members of *Micrococcus* sp. were isolated from the midgut of corn borer *Ostrinia nubilalis* and its cellulase, xylanase and β-glucosidase were quantitatively determined (Vilanova et al., 2012). Several *Staphylococcus* sp. are also cellulolytic (Jaishree et al., 1986; Pourramezan et al., 2012; Manfredi et al., 2015; Ventorino et al., 2015). *Sphingomonas* sp. are frequently found in forest soils (Männistö and Häggblom, 2006; Talia et al., 2012), and has endoglucanase, β-glucosidase and ligninolytic activities (Masai et al., 2007). *Cellulomonas* sp., which are frequently present in the soil (Kang et al., 2007; Kim et al., 2008; Yin et al., 2010; Talia et al., 2012), are capable of growing in sugarcane bagasse (Ponce-Noyola and De La Torre, 2001). Its cellulase activity has been well characterized (Shen et al., 1995; Nikolova et al., 1997; Sánchez-Herrera et al., 2007; Pérez-Avalos et al., 2008; Jing et al., 2009; Maki et al., 2009; Saratale et al., 2010). Members of *Pseudomona* sp. were reported as cellulolytic (Huang et al., 2012; Pourramezan et al., 2012; Nandimath et al., 2016) and β-glucosidase activity was also reported (Tarayre et al., 2014).

We investigated the response of the *A. grandis* gut microbiota to different lignocellulosic diets (varying in fiber and protein content). The higher endoglucanase, xylanase and β-glucosidase activities were observed in larvae grown in CM, followed by NG and finally by CS. These findings can be explained by the recalcitrance nature of a residue like CS caused by its complex lignocellulosic content. The highest enzymatic activities occurred in a pH range between 5 and 6, except for endoglucanase activity that had two optimal pH values, 5 and 7. We also observed that the optimal temperature was 50°C in all assays. These results are in agreement with insect cellulases characterized by other authors, who reported the highest activities at similar temperature and pH levels (Lee et al., 2004; Byeon et al., 2005; Wei et al., 2006; Kim et al., 2008; Willis et al., 2011; Xia et al., 2013).

Previous studies assessed the effect on cellulase activity in the guts of termites and beetles due to changes in the diet (Geib et al., 2009; Li et al., 2013). Geib et al. (2009) studied the effect of the diets on the gut of larval Longhorned beetle, *Anoplophora glabripennis*. They demonstrated that larvae fed on wood from a resistant tree (*Pyrus calleryana*) showed no cellulase activity, whereas larvae fed on preferred tree (*Acer ssacharum*) had a high enzymatic activity. Also, they proposed a direct correlation between bacterial community diversity, which is determined by diets, and gut cellulase activity.

The high-throughput analysis of the 16S rRNA amplicons demonstrated that changes in diet influences the composition of the microbial community present in the *A. grandis* gut. These results contribute to answer the question of whether changes in the abundance of cellulose degrading microbiota contribute to change overall cellulolytic activity.

This type of study can contribute to a more complete characterization of the insect’s cellulolytic processes and to the discovery of novel and more efficient lignocellulosic enzymes; which could help to reduce the high cost in bioethanol industry.

## Author Contributions

Conceived and designed the experiments: EGB, JC-N, and PT. Performed the experiments: EGB, RS, and PT. Analyzed the data: EGB, MS, and PT. Contributed reagents/materials/analysis tools: RS, MS, JC-N, EC, EGB, and PT. Wrote the manuscript: EGB, MS, and PT. Contributed to the critical revision of the manuscript: JC-N, and EGB.

## Conflict of Interest Statement

The authors declare that the research was conducted in the absence of any commercial or financial relationships that could be construed as a potential conflict of interest.

## Footnotes

1 http://www.arb-silva.de/

2 http://www.mothur.org/wiki/Silva_reference_files
