# Supplementary material for: Effect of Different Lignocellulosic Diets on Bacterial Microbiota and Hydrolytic Enzyme Activities in the Gut of the Cotton Boll Weevil (Anthonomus grandis)
Source: Front Microbiol. 2016 Dec 27;7:2093. doi: 10.3389/fmicb.2016.02093 (PMC5186755; doi:10.3389/fmicb.2016.02093)
Supplement: Supplementary file 1 [file Table_1.DOCX]

**Table S1. Distribution of shared phylotypes in replicate batches of cotton boll weevils fed in CM, CS and PP artificial diets.**

| **OTUs** | **Phylum** | **Family** | **Genus** |
| --- | --- | --- | --- |
| **Cottonseed meal (CM)** | | | |
| 0001 | Proteobacteria | Enterobacteriaceae | unclassified |
| 0002 | Proteobacteria | Pseudomonadaceae | *Pseudomonas* sp. |
| 0004 | Proteobacteria | Comamonadaceae | *Delftia* sp. |
| 0006 | Firmicutes | Staphylococcaceae | *Staphylococcus* sp*.* |
| 0007 | Firmicutes | Paenibacillaceae | *Fontibacillus* sp*.* |
| 0008 | Proteobacteria | Xanthomonadaceae | *Stenotrophomonas* sp. |
| 0011 | Actinobacteria | Cellulomonadaceae | *Cellulomonas* sp*.* |
| 0014 | Proteobacteria | Moraxellaceae | *Acinetobacter* sp. |
| 0015 | Proteobacteria | Moraxellaceae | *Acinetobacter* sp*.* |
| 0016 | Proteobacteria | Pseudomonadaceae | *Pseudomonas* sp*.* |
| 0018 | Proteobacteria | unclassified | unclassified |
| 0020 | Proteobacteria | Pseudomonadaceae | *Pseudomonas* sp*.* |
| 0022 | Actinobacteria | Micrococcaceae | *Micrococcus* sp. |
| 0024 | Firmicutes | Paenibacillaceae | *Cohnella* sp. |
| 0030 | Firmicutes | Bacillaceae | *Bacillus* sp*.* |
| 0031 | Proteobacteria | Moraxellaceae | *Moraxella* sp. |
| 0032 | Proteobacteria | Hydrogenophilaceae | *Hydrogenophilus* sp. |
| 0041 | Proteobacteria | Pseudomonadaceae | *Pseudomonas* sp*.* |
| 0048 | Proteobacteria | Oxalobacteraceae | *Undibacterium* sp. |
| 0062 | Bacteroidetes | Flavobacteriaceae | *Cloacibacterium sp.* |
| 0071 | Firmicutes | Enterococcaceae | unclassified |
| 0109 | Proteobacteria | unclassified | unclassified |
| 0144 | Proteobacteria | Enterobacteriaceae | unclassified |
| 0170 | Proteobacteria | Enterobacteriaceae | unclassified |
| 0173 | Proteobacteria | unclassified | unclassified |
| 0218 | Proteobacteria | Pseudomonadaceae | unclassified |
| 0291 | Proteobacteria | Enterobacteriaceae | unclassified |
| 0362 | Proteobacteria | Comamonadaceae | unclassified |
|  |  |  |  |
| **Corn stover (CS)** | | | |
| 0001 | Proteobacteria | Enterobacteriaceae | unclassified |
| 0002 | Proteobacteria | Pseudomonadaceae | *Pseudomonas* sp*.* |
| 0003 | Spirochaetes | Spirochaetaceae | *Treponema* sp*.* |
| 0004 | Proteobacteria | Comamonadaceae | *Delftia* sp*.* |
| 0005 | Spirochaetes | Spirochaetaceae | *Treponema* sp*.* |
| 0006 | Firmicutes | Staphylococcaceae | *Staphylococcus* sp*.* |
| 0007 | Firmicutes | Paenibacillaceae | *Fontibacillus* sp*.* |
| 0008 | Proteobacteria | Xanthomonadaceae | *Stenotrophomonas* sp. |
| 0009 | Spirochaetes | Spirochaetaceae | *Treponema* sp*.* |
| 0010 | Spirochaetes | Spirochaetaceae | *Treponema* sp*.* |
| 0011 | Actinobacteria | Cellulomonadaceae | *Cellulomonas* sp*.* |
| 0012 | Fibrobacteres | Fibrobacteraceae | unclassified |
| 0013 | Spirochaetes | Spirochaetaceae | *Treponema* sp*.* |
| 0014 | Proteobacteria | Moraxellaceae | *Acinetobacter* sp*.* |
| 0015 | Proteobacteria | Moraxellaceae | *Acinetobacter* sp*.* |
| 0017 | Spirochaetes | Spirochaetaceae | unclassified |
| 0021 | Spirochaetes | Spirochaetaceae | *Treponema* sp. |
| 0022 | Actinobacteria | Micrococcaceae | *Micrococcus* sp. |
| 0023 | Firmicutes | Streptococcaceae | *Lactococcus* sp*.* |
| 0025 | Proteobacteria | Sphingomonadaceae | *Sphingomonas* sp. |
| 0027 | Spirochaetes | Spirochaetaceae | *Treponema* sp. |
| 0029 | unclassified | unclassified | unclassified |
| 0031 | Proteobacteria | Moraxellaceae | *Moraxella* sp. |
| 0035 | Proteobacteria | unclassified | unclassified |
| 0036 | Proteobacteria | Oxalobacteraceae | *Massilia* sp. |
| 0038 | Firmicutes | Streptococcaceae | *Lactococcus* sp*.* |
| 0043 | Spirochaetes | Spirochaetaceae | *Treponema* sp. |
| 0049 | Spirochaetes | Spirochaetaceae | unclassified |
| 0050 | Fibrobacteres | Fibrobacteraceae | unclassified |
| 0051 | Firmicutes | Streptococcaceae | *Streptococcus sp.* |
| 0053 | Actinobacteria | unclassified | unclassified |
| 0054 | Acidobacteria | Holophagae_unclassified | unclassified |
| 0056 | Fibrobacteres | Fibrobacteraceae | unclassified |
| 0057 | Spirochaetes | Spirochaetaceae | *Treponema* sp. |
| 0058 | Firmicutes | Ruminococcaceae | unclassified |
| 0064 | Fibrobacteres | Fibrobacteraceae | unclassified |
| 0066 | Firmicutes | Ruminococcaceae | unclassified |
| 0068 | unclassified | unclassified | unclassified |
| 0070 | Spirochaetes | Spirochaetaceae | *Treponema* sp. |
| 0071 | Firmicutes | Enterococcaceae | unclassified |
| 0072 | unclassified | unclassified | unclassified |
| 0074 | Proteobacteria | Rhizobiaceae | unclassified |
| 0079 | Spirochaetes | Spirochaetaceae | *Treponema* sp. |
| 0080 | Firmicutes | Family XI | *Anaerococcus* sp. |
| 0081 | unclassified | unclassified | unclassified |
| 0082 | Firmicutes | Family XIIII | unclassified |
| 0083 | Proteobacteria | Desulfovibrionaceae | *Desulfovibrio* sp. |
| 0084 | Spirochaetes | Spirochaetaceae | unclassified |
| 0085 | Bacteroidetes | unclassified | unclassified |
| 0088 | Bacteroidetes | Porphyromonadaceae | *Tannerella sp.* |
| 0089 | Bacteroidetes | unclassified | unclassified |
| 0096 | Fibrobacteres | unclassified | unclassified |
| 0100 | Bacteroidetes | Porphyromonadaceae | *Tannerella sp.* |
| 0102 | Chlorobi | unclassified | unclassified |
| 0104 | Planctomycetes | Planctomycetaceae | unclassified |
| 0105 | Bacteroidetes | unclassified | unclassified |
| 0106 | unclassified | unclassified | unclassified |
| 0110 | unclassified | unclassified | unclassified |
| 0113 | Firmicutes | Lachnospiraceae | unclassified |
| 0117 | Spirochaetes | Spirochaetaceae | *Treponema* sp. |
| 0121 | unclassified | unclassified | unclassified |
| 0122 | Proteobacteria | Desulfovibrionaceae | unclassified |
| 0128 | Spirochaetes | Spirochaetaceae | *Treponema* sp. |
| 0129 | Firmicutes | Ruminococcaceae | unclassified |
| 0130 | Firmicutes | Ruminococcaceae | unclassified |
| 0131 | Spirochaetes | Spirochaetaceae | *Treponema* sp. |
| 0132 | Spirochaetes | Spirochaetaceae | *Treponema* sp. |
| 0133 | Bacteroidetes | Porphyromonadaceae | unclassified |
| 0138 | Firmicutes | Ruminococcaceae | unclassified |
| 0139 | Bacteroidetes | unclassified | unclassified |
| 0141 | Firmicutes | Family XIII | unclassified |
| 0142 | Actinobacteria | unclassified | unclassified |
| 0143 | Spirochaetes | Leptospiraceae | unclassified |
| 0144 | Proteobacteria | Enterobacteriaceae | unclassified |
| 0147 | Spirochaetes | Spirochaetaceae | *Treponema* sp. |
| 0148 | Bacteroidetes | unclassified | unclassified |
| 0150 | unclassified | unclassified | unclassified |
| 0154 | unclassified | unclassified | unclassified |
| 0157 | Firmicutes | Lachnospiraceae | unclassified |
| 0159 | Spirochaetes | Spirochaetaceae | *Treponema* sp. |
| 0166 | Firmicutes | Family XIII | unclassified |
| 0167 | Bacteroidetes | unclassified | unclassified |
| 0168 | Firmicutes | Family XIII | unclassified |
| 0169 | unclassified | unclassified | unclassified |
| 0174 | Firmicutes | Family XI | *Peptoniphilus* sp. |
| 0185 | Spirochaetes | Spirochaetaceae | *Treponema* sp. |
| 0189 | Firmicutes | Ruminococcaceae | unclassified |
| 0193 | Proteobacteria | Methylophilaceae | *Methylophilus* sp. |
| 0194 | Firmicutes | Syntrophomonadaceae | unclassified |
| 0195 | Firmicutes | unclassified | unclassified |
| 0200 | Firmicutes | unclassified | unclassified |
| 0201 | Firmicutes | Peptococcaceae | unclassified |
| 0202 | Bacteroidetes | Cytophagaceae | Spirosoma sp. |
| 0203 | Firmicutes | Ruminococcaceae | unclassified |
| 0204 | Spirochaetes | Spirochaetaceae | unclassified |
| 0206 | Firmicutes | Christensenellaceae | unclassified |
| 0212 | Bacteroidetes | unclassified | unclassified |
| 0225 | Bacteroidetes | unclassified | unclassified |
| 0229 | Spirochaetes | Spirochaetaceae | *Treponema* sp. |
| 0233 | Firmicutes | Clostridiaceae_1 | unclassified |
| 0234 | Synergistetes | Synergistaceae | unclassified |
| 0238 | Bacteroidetes | unclassified | unclassified |
| 0240 | unclassified | unclassified | unclassified |
| 0241 | Firmicutes | Bacillaceae | unclassified |
| 0243 | unclassified | unclassified | unclassified |
| 0244 | unclassified | unclassified | unclassified |
| 0246 | Firmicutes | Ruminococcaceae | *Ruminococcus* sp. |
| 0248 | unclassified | unclassified | unclassified |
| 0252 | Cyanobacteria | unclassified | unclassified |
| 0256 | Fibrobacteres | unclassified | unclassified |
| 0260 | Spirochaetes | Spirochaetaceae | *Treponema* sp. |
| 0261 | Spirochaetes | Spirochaetaceae | *Treponema* sp. |
| 0263 | Bacteroidetes | unclassified | unclassified |
| 0264 | Planctomycetes | Planctomycetaceae | unclassified |
| 0265 | Proteobacteria | Acetobacteraceae | *Acidicaldus* sp. |
| 0270 | Firmicutes | Christensenellaceae | unclassified |
| 0271 | Firmicutes | Ruminococcaceae | *Anaerotruncus* sp. |
| 0273 | Firmicutes | Peptococcaceae | *Dehalobacterium* sp. |
| 0281 | Fibrobacteres | unclassified | unclassified |
| 0287 | unclassified | unclassified | unclassified |
| 0300 | Tenericutes | Mollicutes_RF9_unclassified | unclassified |
| 0304 | unclassified | unclassified | unclassified |
| 0305 | unclassified | unclassified | unclassified |
| 0306 | unclassified | unclassified | unclassified |
| 0308 | Parcubacteria | unclassified | unclassified |
| 0309 | Bacteroidetes | unclassified | unclassified |
| 0316 | unclassified | unclassified | unclassified |
| 0321 | unclassified | unclassified | unclassified |
| 0322 | Tenericutes | Mollicutes_RF9_unclassified | unclassified |
| 0325 | unclassified | unclassified | unclassified |
| 0328 | Actinobacteria | Nocardiaceae | *Rhodococcus* sp. |
| 0341 | Bacteroidetes | Rikenellaceae | unclassified |
| 0343 | Firmicutes | Lactobacillales_unclassified | unclassified |
| 0372 | Actinobacteria | unclassified | unclassified |
| 0401 | unclassified | unclassified | unclassified |
| 0406 | Proteobacteria | unclassified | unclassified |
| 0425 | Firmicutes | Ruminococcaceae | unclassified |
| 0432 | Spirochaetes | Spirochaetaceae | *Treponema* sp*.* |
| 0436 | Spirochaetes | Spirochaetaceae | unclassified |
| 0445 | Firmicutes | Peptococcaceae | *Sporotomaculum* sp. |
|  |  |  |  |
| **Napier grass (NG)** | | | |
| 0001 | Proteobacteria | Enterobacteriaceae | unclassified |
| 0002 | Proteobacteria | Pseudomonadaceae | *Pseudomonas* sp*.* |
| 0003 | Spirochaetes | Spirochaetaceae | *Treponema* sp*.* |
| 0004 | Proteobacteria | Comamonadaceae | *Delftia* sp*.* |
| 0006 | Firmicutes | Staphylococcaceae | *Staphylococcus* sp*.* |
| 0007 | Firmicutes | Paenibacillaceae | *Fontibacillus* sp. |
| 0008 | Proteobacteria | Xanthomonadaceae | *Stenotrophomonas* sp. |
| 0011 | Actinobacteria | Cellulomonadaceae | *Cellulomonas* sp*.* |
| 0014 | Proteobacteria | Moraxellaceae | *Acinetobacter* sp. |
| 0015 | Proteobacteria | Moraxellaceae | *Acinetobacter* sp. |
| 0018 | Proteobacteria | unclassified | unclassified |
| 0022 | Actinobacteria | Micrococcaceae | *Micrococcus* sp. |
| 0032 | Proteobacteria | Hydrogenophilaceae | *Hydrogenophilus* sp. |
| 0033 | Firmicutes | Bacillaceae | *Geobacillus sp.* |
| 0036 | Proteobacteria | Oxalobacteraceae | *Massilia* sp. |
| 0039 | Actinobacteria | Intrasporangiaceae | unclassified |
| 0044 | Proteobacteria | Caulobacteraceae | *Brevundimonas* sp. |
| 0046 | Actinobacteria | unclassified | unclassified |
| 0055 | Actinobacteria | Corynebacteriaceae | *Corynebacterium sp.* |
| 0060 | Firmicutes | unclassified | unclassified |
| 0092 | Firmicutes | Streptococcaceae | *Streptococcus sp.* |
| 0144 | Proteobacteria | Enterobacteriaceae | unclassified |
| 0173 | Proteobacteria | unclassified | unclassified |
| 0214 | Proteobacteria | Enterobacteriaceae | unclassified |
